# Errata

**Published:** 1856-01

**Authors:** 


ERRATA.

This number should have been dated January and February. 1856

